# Concordance between clinical diagnoses and autopsies anatomopathological findings at a tertiary-level university hospital

**DOI:** 10.7705/biomedica.7097

**Published:** 2024-11-06

**Authors:** Julieth Alexandra Franco, Mario Miguel Barbosa, César Redondo

**Affiliations:** 1 Departamento de Patología, Universidad Remington, Medellín, Colombia Departamento de Patología Universidad Remington Medellín Colombia; 2 Centro de Investigaciones Clínicas, Fundación Valle del Lili, Cali, Colombia Fundación Valle del Lili Cali Colombia; 3 Facultad de Ciencias de la Salud, Universidad ICESI, Cali, Colombia Universidad Icesi Facultad de Ciencias de la Salud Universidad ICESI Cali Colombia; 4 División de Traumatología y Cirugía de Cuidados Intensivos, Departamento de Cirugía, Universidad del Valle, Cali, Colombia Universidad del Valle Departamento de Cirugía Universidad del Valle Cali Colombia; 5 Departamento de Patología, Universidad de Cartagena, Cartagena de Indias, Colombia Universidad de Cartagena Departamento de Patología Universidad de Cartagena Cartagena Colombia

**Keywords:** Autopsy, diagnosis, pathology, autopsia, diagnóstico, patología

## Abstract

**Introduction.:**

A clinical autopsy is a fundamental diagnostic tool for confirming the diagnosis of diseases of public health interest. However, the clinical-pathological concordance has not been evaluated.

**Objective.:**

To determine the concordance between clinical diagnoses and anatomopathological findings from autopsies conducted at a tertiary-level hospital institution.

**Materials and methods.:**

A descriptive, observational, cross-sectional, and retrospective study where we analyzed autopsy cases performed between 2015 and 2019. The variables studied were age, gender, origin, and clinical and anatomopathological diagnoses.

**Results.:**

The concordance degree was slight (k = 0.30; 95% CI: 0.21-0.42), which coincides with internationally reported findings in healthcare institutions with a similar patient population and availability of diagnostic resources. The clinical-pathological discrepancy, as evidenced according to the Goldman *et al.* classification, then modified by Battle *et al.,* was 57.3% (47/82), corresponding to major discrepancies, a value within the expected limits.

**Conclusions.:**

The concordance between clinical diagnoses and anatomopathological findings in autopsies is slight, and the discrepancies are within the expected range. This study highlights the importance of improving clinical and syndromic diagnosis of public health interest.

## Abstract (es)

**Introducción.:**

La autopsia clínica es una ayuda fundamental para confirmar el diagnóstico de enfermedades de interés en salud pública. Sin embargo, la concordancia clínico-patológica no ha sido evaluada.

**Objetivo.:**

Determinar la concordancia entre los diagnósticos clínicos y los hallazgos anatomopatológicos de autopsias en una institución hospitalaria de tercer nivel.

**Materiales y métodos.:**

Se desarrolló un estudio descriptivo, observacional, transversal y retrospectivo, en el cual se analizaron las autopsias practicadas entre el 2015 y el 2019. Las variables estudiadas fueron: edad, sexo, procedencia, diagnósticos clínicos y hallazgos anatomopatológicos.

**Resultados.:**

El grado de concordancia fue leve (k = 0,30; IC_95%_: 0,21-0,42), lo cual coincide con lo reportado internacionalmente en instituciones de salud con una población de pacientes y disponibilidad de recursos diagnósticos similares. La discrepancia clínico-patológica evidenciada según la clasificación de Goldman *et al.,* modificada por Battle *et al.,* fue del 57,3 % (47/82) correspondientes a discrepancias mayores, valor que se encuentra dentro de los limites esperados.

**Conclusiones.:**

La ligera concordancia clínico-patológica de las autopsias clínicas y las discrepancias se encuentran dentro del rango esperado. Lo anterior resalta la importancia de mejorar el diagnóstico clínico y sindromático de las enfermedades de interés en salud pública.

The autopsy procedure has a history of 2,000 years and has transcended the boundaries of time, becoming a traditional tool for pathologists in their daily work. The word "autopsy" etymologically derives from the Greek term "autopsía" (αὐτοψία), which means "action of seeing with one's own eyes". This process implies a systemic and multidisciplinary evaluation combined with a series of scientific procedures that consist of two stages: the external anatomical examination of the body and the meticulous analytical examination of the internal cavities and organs ^1^. In Colombia, autopsies are classified as forensic and clinical. A forensic autopsy is performed for judicial investigation purposes, while clinical autopsies encompass all other cases depending on their specific objectives:

- sanitary autopsies are conducted to address public health concerns and interests.
- educational autopsies aim to enhance teaching and learning processes, providing valuable insights for medical education, and,
- investigative autopsies are pursued to advance scientific research objectives in pure or applied contexts.

According to the American College of Pathology, clinical autopsies are indicated in various circumstances, including cases where the autopsy can provide relevant information for the family or the public, such as in epidemiological surveillance. The practice of a clinical autopsy is widely recognized as an invaluable tool for medical and surgical education and a reliable indicator of healthcare quality. It is considered the gold standard for evaluating medical procedures ^2^. Additionally, it plays a crucial role in assessing the quality of premortem medical care and improving the accuracy of data recording and management in hospital statistics. This method empowers healthcare administrators to implement necessary measures to enhance the quality of medical practice, particularly in the context of diseases under epidemiological surveillance ^3^^-^^6^.

Currently, medical and diagnostic evaluations are based on technological advancements in clinical laboratories and imaging, supported by semiotic assessment and clinical algorithms as the main diagnostic methods. However, these procedures have demonstrated limitations related to the most relevant function of autopsies: clinical-pathological correlation. Multiple international and national studies have shown the discordance between clinical diagnoses and postmortem findings ^2^^,^^7^. Autopsy reports provide an additional criterion for determining causes of death and reveal anatomopathological alterations that were unsuspected during medical management. This scenario highlights the inherent uncertainty in clinical diagnoses and healthcare services ^3^.

Clinical-pathological discrepancies are measured by the Goldman *et al.* classification, which was modified by Battle *et al.* It categorizes discrepancies as major, referring to preexisting major diseases and primary causes of death; and minor, defined as preexisting illnesses, related diagnoses, contributing causes, or other significant clinical conditions. These categories are further subdivided into classes I and II for major discrepancies and classes III and IV for minor discrepancies. Each classification is based on clinical criteria and the medical management established for each case ^5^^,^^8^.

The rates of significant discrepancies between clinical and anatomopathological diagnoses in autopsies for adult and pediatric populations have ranged from 10 to 30% of all autopsy cases ^5^. Previously, Cabot compared clinical diagnoses with findings in 1,000 autopsies and found that 40% were inaccurate or incorrect ^9^. In other studies, reported discrepancies in adults range from 4.1% to 39% for class I and 11.2% to 19.2% for class II, according to Goldman's criteria ^8^^,^^10^. Mexico, Costa Rica, and Honduras have reported similar results to those found in the international literature in pediatric, mother-child, and adult populations ^10^^,^^11^.

In 2004, the *Hospital Universitario del Valle Evaristo García E.S.E.,* in Cali, conducted an evaluation in two periods, from 1970 to 1975 and 1990 to 1995, involving 200 autopsies. The study found clinical diagnosis confirmation of the underlying cause of death in 73% (1970) and 64% (1990) of the autopsies. However, the terminal cause of death was confirmed only during the autopsy in 34% and 38% of the cases, respectively. The most diagnosed clinical conditions leading to death were cardiogenic and septic shock ^2^.

In 2015, a study evaluated the agreement between clinical diagnoses and anatomopathological findings in children who died from pneumonia in the pediatric intensive care units of two institutions in Bogotá. The study revealed that clinical history and complementary tests had an error rate of up to 30%. There was a poor correlation between clinical diagnoses of pneumonia and autopsy findings, primarily due to the potential confusion with other causes of lung involvement ^12^.

The objective of this study was to establish the concordance between the clinical diagnosis and the anatomopathological findings in autopsies performed during the 2015-2019 period at the *Hospital Universitario del Caribe* in Cartagena.

## Materials and methods

A descriptive retrospective cross-sectional study was conducted to review autopsy reports and medical records of patients who underwent clinical autopsy protocols at the Pathology Laboratory of the *Hospital Universitario del Caribe* in Cartagena between 2015 and 2019. We included medical records of patients older than one year who underwent clinical autopsy protocols during the specified period, with documented primary clinical diagnosis and without reported autolytic changes in the autopsy reports. The reviewed medical records included age, gender, place of origin (municipality, neighborhood), and clinical diagnoses and anatomopathological findings. These diagnoses were classified according to the tenth revision of the International Classification of Diseases (ICD-10).

### Estimation of clinical-pathological discrepancy

To determine clinical-pathological discrepancies, we used the modified Goldman's classification, which defines them as major anatomopathological diagnoses related to preexisting major diseases and primary causes of death ^5^^,^^11^; and minor anatomopathological diagnoses associated with preexisting illnesses, related diagnoses, contributing causes, or other significant conditions.

Major anatomopathological diagnoses are subdivided into classes I and II, whereas minor anatomopathological diagnoses are subclassified into class III and IV:

- *Class I:* Discordance in major diagnoses where detection during life could have altered the treatment and likely prolonged life or led to a cure. The diagnosis was not reached due to a lack of clinical suspicion, inconclusive, confusing, or incorrectly interpreted diagnostic test results, or unavailability of diagnostic test results.
- *Class II:* Discordance in major diagnoses where detection during life would not have modified the treatment due to a lack of specific treatment, patient entering cardiac arrest that does not respond to appropriate cardiopulmonary resuscitation maneuvers, patient correctly treated even though the diagnosis was unknown, or patient or family refusal of diagnostic or therapeutic measures.
- *Class III:* Discordance in minor diagnoses related to terminal illness but not directly related to death.
- *Class IV:* Discordance in minor diagnoses that eventually affect the prognosis or processes contributing to death in patients with terminal illness.

### Statistical analysis

A descriptive analysis was performed for qualitative variables, including univariate and bivariate analyses with absolute and relative frequencies. For continuous variables, the arithmetic mean was used as the central tendency measure, and the standard deviation was used as a measure of dispersion.

### Estimation of concordance

Concordance between clinical and anatomopathological diagnoses was estimated using the kappa index, prevalence, overall agreement, and positive and negative agreement. All estimates are reported with 95% confidence intervals. Despite convenience sampling, we calculated the required sample size to achieve 80% power and 95% confidence for the obtained kappa. Analyses were performed using R, version 4.0, with the kappa size and packages.

In this approach, an autopsy is considered an additional evaluation of the studied phenomenon rather than a perfect gold standard. As Barnhart ^13^ notes, the concept of absolute agreement is more appropriate than validity when comparing methods that measure the same construct on the same scale, and perfect agreement means identical measurements. Validity measures such as sensitivity and specificity assume one method represents the "truth" against which the other is compared.

However, in this study, clinical diagnosis and autopsy are subject to error or interpretation variability. Therefore, the aim is to quantify the agreement between methods rather than validate clinical diagnosis against autopsy ^13^^,^^14^. Kappa, prevalence, overall agreement, and positive and negative agreement provide a more comprehensive assessment of agreement than a single coefficient ^13^. The precision of estimates (95% CI) and adequate sample size are reported, following current recommendations for rigorous agreement evaluation ^13^.

### Ethical aspects

The present investigation was classified as without risk, according to Resolution 8430 of 1993 from the Colombian ministry of Health. It was a descriptive retrospective study that involved the analysis and comparison of medical records and autopsy reports. To ensure confidentiality, the patients' identities, treating physicians, and healthcare institutions remained anonymous. The d ata collected were accessed and used solely by the researchers of this study. Furthermore, the researchers strictly adhered to the principles outlined in the Helsinki Declaration, which governs medical research involving human subjects. The protocol of this study was approved by the ethics committee of the *Hospital Universitario del Caribe.*

## Results

The Pathology Department of the *Hospital Universitario del Caribe* performed 136 autopsies from 2015 to 2019. Out of these, 82 autopsies met the inclusion criteria. The distribution by year is as follows: 11 autopsies in 2015, 12 in 2016, 16 in 2017, 13 in 2018, and 30 in 2019. Autopsies on individuals under 18 years old accounted for 36 cases, while autopsies on individuals over 18 years old accounted for 46 cases. The gender distribution was similar, with 36 cases involving males and 46 cases involving females.

The municipality of Cartagena accounted for most cases, with 65 (79.27%), followed by Villanueva with 5 (6.1%) (table 1). Out of the 205 neighborhoods in the city of Cartagena, 34 were reported. Among them, El Pozón and La Playa had 6 cases each (7.3%), and San José de los Campanos had 5 (6.1%).

**Table 1:** Distribution of clinical diagnoses and anatomopathological findings of causes of death by age and sex

Category	Age			Sex			p value
	Adults	Children	p	Female	Male	Total	
	(≥ 18 years)	(< 18 years)	value	n (%)	n (%)		
	n (%)	n (%)					
Clinical diagnoses							
Circulatory	8 (17.4)	1 (2.8)		6 (13.0)	3 (8.3)	9 (11.0)	
Digestive	3 (6.5)	1 (2.8)		1 (2.2)	3 (8.3)	4 (4.9)	
Central nervous system	3 (6.5)	2 (5.6)		5 (10.9)	0 (0.0)	5 (6.1)	
Respiratory	4 (8.7)	7 (19.4)		5 (10.9)	6 (16.7)	11 (13.4)	
Skin and subcutaneous tissue	1 (2.2)	0 (0.0)		0 (0.0)	1 (2.8)	1 (1.2)	
Infectious diseases	15 (32.6)	14 (38.9)		15 (32.6)	14 (38.9)	29 (35.4)	
Pregnancy, childbirth, and the puerperium-related	1 (2.2)	0 (0.0)		1 (2.2)	0 (0.0)	1 (1.2)	
Symptoms, signs, and abnormal clinical findings, not classified elsewhere	10 (21.7)	7 (19.4)		10 (21.7)	7 (19.4)	17 (20.7)	
Tumors/hematopoietic diseases	1 (2.2)	4 (11.1)	0.19	3 (6.5)	2 (5.6)	5 (6.1)	0.36
Anatomopathological findings							
Circulatory	7 (15.2)	7 (19.4)	0.49	6 (13.0)	8 (22.2)	14 (17.1)	0.24
Digestive	5 (10.9)	2 (5.6)		3 (6.5)	4 (11.1)	7 (8.5)	
Central nervous system	5 (10.9)	1 (2.8)		4 (8.7)	2 (5.6)	6 (7.3)	
Respiratory	3 (6.5)	2 (5.6)		5 (10.9)	0 (0.0)	5 (6.1)	
Infectious diseases	17 (37.0)	18 (50.0)		19 (41.3)	16 (44.4)	35 (42.7)	
Pregnancy, childbirth, and the puerperium-related	0 (0)	0 (0)		0 (0)	0 (0)	0 (0)	
Symptoms, signs, and abnormal clinical findings, not classified elsewhere	3 (6.5)	0 (0.0)		3 (6.5)	0 (0.0)	3 (3.7)	
Tumors/hematopoietic diseases	6 (13.0)	6 (16.7)		6 (13.0)	6 (16.7)	12 (14.6)	

The category of infectious diseases (including parasites) had the highest frequency of reported clinical diagnoses (n = 29, 35.3%), followed by symptoms, signs, and abnormal clinical findings not elsewhere classified (n = 17, 20.7%), and respiratory system pathologies (n = 11, 13.4%). Infectious diseases were the most frequently reported anatomopathological findings (n = 35, 42.68%), followed by circulatory system pathologies (n = 14, 17%), tumors/hematological diseases (n = 12, 14.6%) and symptoms, signs, and abnormal clinical findings not elsewhere classified (n = 3, 3.6%).

Both age groups had the highest frequency of reported diagnosis in the category of infectious diseases (n = 14, 38.9% in individuals under 18 years; n = 15, 32.6% in individuals over 18 years), followed by respiratory pathologies for individuals under 18 years (n = 7, 19.4%) and symptoms, signs, and abnormal clinical findings for individuals over 18 years (n = 10, 21.7%). Regarding gender, males and females showed a similar pattern, with infectious diseases being the most frequent diagnosis (n = 15, 32.6% in females; n = 14, 38.9% in males), followed by the category of symptoms, signs, and abnormal clinical findings (n = 10, 21.7% in females; n = 7, 19.4% in males).

The frequency of anatomopathological findings by age group revealed a higher incidence of infectious diseases in both groups (n = 18, 50% in individuals under 18 years; n = 17, 37.0% in individuals over 18 years), followed by circulatory system pathologies (n = 7, 19.4% for individuals under 18 years; n = 7, 15.2% for individuals over 18 years). Concerning gender, a similar pattern was observed in both groups, with a higher prevalence of infectious diseases (n = 19, 41.3% in females; n = 16, 44.4% in males), followed by circulatory system pathologies (n = 6, 13.0% in females; n = 8, 22.2% in males) (table 1).

The autopsy confirmed the direct cause of death in 35 cases (42.6%), yielding an overall kappa value of 0.30 (95% CI: 0.21 - 0.42). In the category of infectious diseases, we obtained a kappa value of 0.44 (95% CI: 0.34 -0.54). This diagnosis had a prevalence of 43% (95% CI: 32% - 54%), with a 66% (95% CI: 51% - 77%) agreement among positives, and a 78% (95% CI: 68% - 86%) agreement among negatives. The overall agreement percentage was 73% (95% CI: 62% - 82%). Additionally, the number of patients included in the study surpassed the minimum required for this estimation to achieve a power of 80% and a confidence level of 95%, corresponding to a type I error of 5% and a type II error of 20% (tables 2 and 3).

**Table 2:** Correlation degree between clinical diagnoses and anatomopathological findings of causes of death by category

Clinical diagnoses		Anatomopathological findings		Total
		Infectious diseases	Other category	n (%)
		n (%)	n (%)	
Infectious diseases		21 (25.6)	8 (9.8)	29 (35.4)
	Other category	14 (17.1)	39 (47.6)	53 (64.6)
	Total	35 (42.7)	47 (57.3)	82 (100.0)
		Digestive	Other category	Total
Digestive		3 (3.7)	1 (1.2)	4 (4.9)
	Other category	4 (4.9)	74 (90)	78 (95.1)
	Total	7 (8.5)	75 (91)	82 (100.0)
		Circulatory	Other category	Total
Circulatory		4 (4.9)	5 (6.1)	9 (11.0)
	Other category	10 (12.2)	63 (76.8)	73 (89.0)
	Total	14 (17.1)	68 (82.9)	82 (100.0)
		Central nervous system	Other category	Total
Central nervous system		1 (1.2)	4 (4.9)	5 (6.1)
	Other category	5 (6.1)	72 (87.8)	77 (93.9)
	Total	6 (7.3)	76 (92.7)	82 (100.0)
		Respiratory system	Other category	Total
Respiratory		3 (3.7)	8 (9.8)	11 (13.4)
	Other category	2 (2.4)	69 (84.1)	71 (86.6)
	Total	5 (6.1)	77 (93.9)	82 (100)

**Table 3:** Kappa and agreement percentages

Category	Kappa index	Prevalence	Agreement	Positive agreement	Negative agreement	MNP
	(95% CI)	(95% CI)	% (95% CI)	% (95% CI)	% (95% CI)	
Circulatory	0.25 (0.11 - 0.39)	0.17 (0.1 - 0.27)	0.82 (0.72 - 0.89)	0.35 (0.13 - 0.59)	0.89 (0.94 - 0.94)	136
Digestive	0.52 (0.33 - 0.71)	0.09 (0.04 - 0.17)	0.94 (0.86 - 0.98)	0.55 (0.19 - 0.82)	0.97 (0.93 - 0.99)	30
Central nervous system	0.12 (-0.04 - 0.28)	0.07 (0.03 - 0.15)	0.89 (0.8 - 0.95)	0.18 (0.01 - 0.52)	0.94 (0.89 - 0.97)	649
Respiratory	0.32 (0.16 - 0.48)	0.06 (0.02 - 0.14)	0.88 (0.79 - 0.94)	0.38 (0.12 - 0.65)	0.93 (0.88 - 0.97)	82
Infectious diseases	0.44 (0.34 - 0.54)	0.43 (0.32 - 0.54)	0.73 (0.62 - 0.82)	0.66 (0.51 - 0.77)	0.78 (0.68 - 0.86)	42
Symptoms, signs, and abnormal clinical findings, not classified elsewhere	0.15 (0.04 - 0.26)	0.04 (0.01 - 0.1)	0.8 (0.7 - 0.88)	0.2 (0.04 - 0.46)	0.89 (0.93 - 0.93)	400
Tumors/hematopoietic diseases	0.03 (-0.08 - 0.14)	0.15 (0.08 - 0.24)	0.82 (0.72 - 0.89)	0.12 (0.01 - 0.38)	0.9 (0.84 - 0.94)	19,620

MNP: Minimum number of patients

In the category of respiratory system diseases, we obtained a kappa value of 0.32 (95% CI: 0.16 - 0.48), confirming a prevalence of 6% (95% CI: 2% - 14%). The agreement percentage among positives was 38% (95% CI: 12% - 65%), while among negatives, it reached 93% (95% CI: 88% - 97%). Overall, the percentage of the agreement was 88% (95% CI: 79% - 94%). In the category of gastrointestinal diseases, we achieved a kappa value of 0.52 (95% CI: 0.33 - 0.71). The prevalence of this diagnosis was 9% (95% CI: 4% - 17%). The percentage among positives was 55% (95% CI: 19% - 82%), and among negatives, it reached 97% (95% CI: 89% - 97%). Overall, the agreement percentage was 94% (95% CI: 86% - 98%).

From the 82 cases, 47 (57.31%) demonstrated a major discrepancy between the premortem clinical diagnosis and postmortem anatomopathological findings. These discrepancies were distributed as follows: 35.3% in class I and 21.9% in class II. No cases were observed in classes III and IV, as underlying diseases or minor related diagnoses were not considered. Only 35 cases (42.68%) showed diagnostic concordance.

## Discussion

Currently, the practice of clinical autopsies is insufficient in our setting, according to the statistics from the public health surveillance of the *Departamento Administrativo Distrital de Salud,* DADIS. In 2015, there were 3,467 reported deaths in the district of Cartagena, with a mortality rate of 346.1 per 100,000 inhabitants ^15^. In the 2015 accountability report of the *Hospital Universitario del Caribe,* the intrahospital mortality rate after 48 hours was 1.94 per 1,000, with only 11 clinical autopsies performed, corresponding to 1.2 per 100,000 inhabitants. In 2019, according to the statistics from the *Departmento Administrativo Nacional de Estadística* (DANE), there were 8,561 deaths in the Bolívar department for individuals over one year old, with a total of 30 autopsies, representing 0.35% ^16^. These figures are significantly lower than those reported in the literature, with approximately 20-30% of annual autopsies ^11^^,^^12^^,^^16^ and even national reports, such as in the Santander department, where an average of 100 autopsies per year is reported. These statistics highlight the underutilization of autopsy over time, raising doubts about its validity and scientific and academic importance ^10^. However, during the evaluated period of this study, autopsies increased by 63%, which may be attributed to circumstances of death, such as patients with diseases of public health interest, unconfirmed diagnoses, or sudden deaths ^17^.

Between 2015 and 2019, autopsy studies predominated in the neonatal and pediatric population, accounting for 89 out of 136 cases (65%). This situation prevails in most healthcare institutions, where autopsies continue to be a crucial procedure in the management of the pediatric population ^2^. In this study population, there was a predominance of adults over 18 years old (46%) and females (46%).

Most cases studied were residents of Cartagena de Indias and the surrounding municipalities in the northern part of the Bolívar department, indicating that the coverage of this service is still centralized and does not allow for a proper assessment of its area of influence. There is no evidence of a significant distribution among the neighborhoods of Cartagena, thus missing the opportunity to evaluate the presence of risk factors associated with the public health diseases reported in this study.

The obtained kappa value for the category of infectious diseases was 0.44, interpreted as moderate agreement. This agreement is driven by concordance between negatives rather than positives. However, the distribution is homogeneous, with a difference of less than 10% between both statistics and an overall agreement of 73%. This estimation is higher than the one reported by Ornellas *et al.,* which corresponded to a kappa value of 33% and included a patient population and diagnostic resource availability similar to those of this study ^18^.

We did not have a kappa value or a positive agreement percentage for infectious diseases in adults when compared with national reports. In the study conducted at the *Hospital Universitario del Valle,* it was reported that "out of 200 adult patients, 62 (31%) had an infectious disease identified during the autopsy, and in 25 (40.3%) of them, it was unknown to the clinicians." In our case, from the 82 patients studied, 35 (42.7%) had an infectious disease reported in the autopsy report, and in 14 (17.1%) of them, it was not clinically suspected ^19^. This result indicates a higher concordance between clinical diagnoses and anatomopathological findings in this category for our study population. However, it is important to emphasize that the *Hospital Universitario del Valle* study used data from populations that differ by more than ten years. This fact could influence the available diagnostic techniques for different infectious diseases. On the other hand, we found a study in a pediatric population who died in an intensive care unit with a clinical diagnosis of pneumonia, in which a negative kappa value of 0.03 was reported, demonstrating the diagnostic difficulty of this pathology in this age group with a degree of agreement interpreted as poor ^12^.

In this category, it is relevant to mention the following findings: Among the 29 patients with clinical suspicion of infectious diseases, 20 appear to have dengue, 8 Zika, and 1 leptospirosis, which was not confirmed. Of the patients suspected of having dengue, 50% (10 out of 20) were confirmed by PCR and three by serology. Additionally, all the Zika cases were confirmed through PCR. Furthermore, histological findings revealed sickle cell disease -an orphan disease- in two confirmed cases, one with dengue and one with Zika. This result highlights the presence of sickle cell disease as a concurrent diagnosis not clinically suspected in these patients ^20^. From a public health perspective, particularly in vector-borne disease surveillance, this finding is important because the physician is who decides when a necropsy should be performed, and specific molecular tests requested. Compared to the kappa value of 0.76, found by Cortés *et al.* for the neoplastic category ^14^, our kappa value was lower (0.03). It is worth mentioning that the *Hospital Universitario del Caribe* does not have an oncology service, so this specific population is less frequently attended to, making the estimation invalid and incomparable.

Similarly, we have the case of the gastrointestinal diseases category, where the kappa value obtained was 0.52, interpreted as moderate concordance. This concordance favors agreement between negatives more than positives, with a difference of 42% between both statistics and an overall concordance of 94%. These findings are consistent with national statistics in which non-neoplastic gastrointestinal pathologies rank as the eighth leading cause of death in the adult population ^21^.

The present study revealed that 57.3% (47/82) of the cases showed a discrepancy between the premortem clinical diagnosis and the postmortem anatomopathological findings. These discrepancies were distributed as 35.3% in class I and 21.9% in class II, with no cases in classes III and IV since underlying diseases or minor related diagnoses were not considered. The percentage of class I is above the accepted average reported in the global literature (10-30%) for major discrepancies, although not exceeding the 40% limit that raises concerns about possible deficiencies in the medical team performance. The levels fall within the accepted range for class II discrepancies, likely because most studied cases occur in the context of unattended deaths or cardiac arrests that do not respond to resuscitation efforts. ^6^^,^^10^^,^^11^^,^^17^^,^^19^^,^^22^^,^^23^.

In terms of strengths, this study determines the sample size and calculates concordance and performance statistics. The degree of diagnostic concordance was assessed using the kappa coefficient. Additionally, the study employs the classification system developed by Goldman *et al.,* then modified by Battle *et al.,* making it the first national study to use this approach. However, there are some limitations to consider. The population size of the study remains relatively small, which may impact on the generalizability of the findings. Furthermore, there is a potential selection bias due to the indications for performing autopsies, which could result in an underestimation or overestimation of the kappa statistic; additionally, the unavailability of death certificates with information about the underlying and direct cause of death as those reported by medical personnel. Despite these challenges, the study provides valuable insights into clinical-pathological discrepancies and contributes to diagnostic accuracy in postmortem examinations ^20^.

## References

[1] Navarro JM (2018). La autopsia clínica. Beneficios relacionados con su práctica. Medisur.

[2] Valdez-Martínez E, Arroyo-Lunagómez E, Landero-López L (1998). Concordancia entre el diagnóstico clínico y el patológico por necropsias. Salud Pública Mex.

[3] Vivas-Moncada S (2016). Caracterización de la autopsia y los hallazgos clínicos en pacientes fallecidos con enfermedad oncológica. Hospital Escuela Dr. Roberto Calderón Gutiérrez, 2007-2015.

[4] Martínez-Portuondo AI, Armas-Pérez L, González-Ochoa E (2007). El diagnóstico por autopsia en Ciudad de La Habana como indicador de la calidad del programa de control de la tuberculosis: 1998-2002. Rev Esp Salud Pública.

[5] Sathirareuangchai S, Shimizu D (2019). Reaffirming the value of the autopsy. Am J Clin Pathol.

[6] Kuijpers CCHJ, Fronczek J, van de Goot FRW, Niessen HWM, van Diest PJ, Jiwa M (2014). The value of autopsies in the era of high-tech medicine: Discrepant findings persist. J Clin Pathol.

[7] Val-Bernal JF (2015). The current role of autopsy in current clinical practice. Med Clin (Barc).

[8] Bürgesser MV, Camps D, Calafat P, Diller A (2011). Discrepancias entre diagnósticos clínicos y hallazgos de autopsia. Rev Argent Anat Patol.

[9] Boada N, Vega L, Centeno M, Siminovich M, Weller G, Sasbón J (2014). Discordancias entre diagnósticos clínicos y anatomopatológicos en 99 pacientes fallecidos en una unidad de cuidados intensivos pediátricos. Rev Argent Ter Intensiva.

[10] Correa YY, Pérez MOB, Moya JAV (2019). Correlación clínico-patológica en fallecidos del Hospital Universitario Clínico Quirúrgico Cmdte. Manuel Fajardo Rivero. Medicentro (Electron).

[11] Valencia-Martínez JE, Rodríguez-Pérez L, Nieto-Andrade B (2006). Discrepancia entre los diagnósticos clínicos y por autopsia en un hospital pediátrico de tercer nivel. Bol Med Hosp Infant Mex.

[12] Castro-Gómez D (2015). Concordancia entre el diagnóstico clínico y los resultados anatomopatológicos de los niños que fallecieron por neumonía en la unidad de cuidado intensivo pediátrico de dos instituciones de la ciudad de Bogotá.

[13] Barnhart HX (2018). A review on assessing agreement. Wiley Stats Ref Stat Ref Online.

[14] Cortés-Reyes É, Rubio-Romero JA, Gaitán-Duarte H (2010). Métodos estadísticos de evaluación de la concordancia y la reproducibilidad de pruebas diagnósticas. Rev Colomb Obstet Ginecol.

[15] Pérez JAD, Uribe MAM (2010). Diseño, realización y evaluación de un sistema de validación del rendimiento del ejercicio médico basado en la autopsia en el Hospital Universitario de Santander 2010. MedUNAB.

[16] Departamento Administrativo Nacional de Estadística (DANE) (2013). Defunciones no fetales.

[17] Baquero-Villa MA (2016). Muerte por establecer y muerte súbita: es cuestión de enfoque. Colomb Forense.

[18] Ornelas-Aguirre JM, Vázquez-Camacho G, González-López L, García-González A, Gámez-Nava JI (2003). Concordance between premortem and postmortem diagnosis in the autopsy: Results of a 10-year study in a tertiary care center. Ann Diagn Pathol.

[19] Botero MP, Carrascal E, Daza Y, Donado P (2004). Concordancia entre el diagnóstico clínico y hallazgos de autopsia en dos períodos en el Hospital Universitario del Valle, Cali. Colomb Med.

[20] Matta L, Barbosa MM, Morales-Plaza CD (2016). Clinical profile of dengue in patients consulting a tertiary hospital in the city of Cali, Colombia, 2013. Biomédica.

[21] Rodríguez-García J, Peñaloza-Quintero RE, Amaya-Lara JL (2017). Estimación de la carga global de enfermedad en Colombia 2012: nuevos aspectos metodológicos. Rev Salud Pública.

[22] Fares AF, Cury PM, Lobo SM (2011). Clinical-pathological discrepancies in critically ill patients with difficult premortem diagnoses. Rev Bras Ter Intensiva.

[23] Tejerina E, Esteban A, Fernández-Segoviano P, Rodríguez-Barbero JM, Gordo F, Frutos-Vivar F (2012). Clinical diagnoses and autopsy findings: Discrepancies in critically ill patients.. Crit Care Med.

